# N6-methyladenosine-methylomic landscape of lung tissues of mice with chronic obstructive pulmonary disease

**DOI:** 10.3389/fimmu.2023.1137195

**Published:** 2023-03-28

**Authors:** Tingting Hu, Lijuan Xu, Min Jiang, Fengbo Zhang, Qifeng Li, Zhiwei Li, Chao Wu, Jianbing Ding, Fengsen Li, Jing Wang

**Affiliations:** ^1^ Xinjiang Laboratory of Respiratory Disease Research, Traditional Chinese Medicine Hospital Affiliated to Xinjiang Medical University, Urumqi, China; ^2^ Fourth Clinical Medical College, Xinjiang Medical University, Ürümqi, China; ^3^ Department of Clinical Laboratory, First Affiliated Hospital of Xinjiang Medical University, Urumqi, China; ^4^ Xinjiang Institute of Pediatrics, Children’s Hospital of Xinjiang Uygur Autonomous Region, Urumqi, China; ^5^ Clinical Laboratory Center, People’s Hospital of Xinjiang Uygur Autonomous Region, Ürümqi, China; ^6^ Department of Respiratory and Critical Care Medicine, People’s Hospital of Xinjiang Uygur Autonomous Region, Ürümqi, China; ^7^ Department of Immunology, School of Basic Medical Science, Xinjiang Medical University, Urumqi, China

**Keywords:** COPD, mouse, lung tissue, m6A, N6-methyladenosine, MeRIP-seq

## Abstract

Chronic obstructive pulmonary disease (COPD), a common respiratory disease, can be divided into stable phase and acute exacerbation phase (AECOPD) and is characterized by inflammation and hyper-immunity. Methylation of N6-methyladenosine (m6A) is an epigenetic modification that regulates the expression and functions of genes by influencing post-transcriptional RNA modifications. Its influence on the immune regulation mechanism has attracted great attention. Herein, we present the m6Amethylomic landscape and observe how the methylation of m6A participates in the pathological process of COPD. The m6A modification of 430 genes increased and that of 3995 genes decreased in the lung tissues of mice with stable COPD. The lung tissues of mice with AECOPD exhibited 740 genes with hypermethylated m6A peak and 1373 genes with low m6A peak. These differentially methylated genes participated in signaling pathways related to immune functions. To further clarify the expression levels of differentially methylated genes, RNA immunoprecipitation sequencing (MeRIP-seq) and RNA-sequencing data were jointly analyzed. In the stable COPD group, 119 hypermethylated mRNAs (82 upregulated and 37 downregulated mRNAs) and 867 hypomethylated mRNAs (419 upregulated and 448 downregulated mRNAs) were differentially expressed. In the AECOPD group, 87 hypermethylated mRNAs (71 upregulated and 16 downregulated mRNAs) and 358 hypomethylated mRNAs (115 upregulated and 243 downregulated mRNAs) showed differential expression. Many mRNAs were related to immune function and inflammation. Together, this study provides important evidence on the role of RNA methylation of m6A in COPD.

## 1 Introduction

Chronic obstructive pulmonary disease (COPD) is a common respiratory disease that causes persistent respiratory symptoms and airflow restriction, owing to airway and/or alveolar abnormalities (1). The World Health Organization survey in 2019 reported 380 million COPD cases in the world and more than 3 million patients annually die from this pathology, which is very alarming (2, 3). The pathogenesis of COPD involves inflammation orchestrated by various inflammatory cells and chemokines. According to clinical symptoms, COPD can be divided into a stable phase and an acute exacerbation phase (AECOPD) (4).

Emerging studies have revealed the role of epigenetic regulation in inflammation, especially the modification of RNA methylation regulatory factors. N6-methyladenosine (m6A) methylation, the most common and abundant post-transcriptional modification of eukaryotic RNA, is dynamically reversible and regulated by methyltransferase, demethylase, and methylated reading protein in eukaryotic cells (5). The methylation process of m6A is mainly regulated by methyltransferases METTL3 and METTL14 that modify m6A by catalyzing adenylate on the mRNA. The demethylase FTO can demethylate the m6A-modified base. The methylation reading proteins YTHDF and YTHDC activate the downstream regulatory pathway by recognizing the base where m6A occurs (6). It was found that m6A was specifically modified at the RRACH site where R is adenine and guanine, H is adenine, cytosine, and uracil, and A is an m6A sequence (7–9). Meanwhile, m6A modification is also related to inflammation in alcoholic kidney injury, coronary artery disease, spontaneous colitis, and other diseases (10–12). A few studies have analyzed the relationship between m6A methylation and COPD. To study the differences in the m6A modification mode between a healthy and COPD model and further explore the relationship between m6A modification and lung tissue inflammation disorder, herein we determine differentially methylated mRNA transcripts in COPD and normal mouse lungs by methylated RNA immunoprecipitation sequencing (MeRIP-seq) and RNA sequencing (RNA-seq). In addition, we analyze the expression of differentially methylated mRNAs in combination with MeRIP-seq and RNA-seq data to investigate if mRNA methylation affects the corresponding gene expression. Overall, this study reveals the potential mechanism of m6A methylation in COPD.

## 2 Materials and methods

### 2.1 Animals and tissue collection

Wild-type C57BL/6J male mice (6-8 weeks old) were purchased from Animal Center of Xinjiang Medical University (China). Mice were housed in a specific pathogen-free environment at Xinjiang Medical University Animal Experimentation Facility under a 12h light/12h dark cycle at 23 ± 1°C and 50 ± 10% relative humidity, fed a non-purified diet and drinking water ad libitum. The study was approved by the Institutional Review Board and Animal Experimentation Committee of Xinjiang Medical University (license number: SYXK-2018-0003) and conducted in accordance with the Xinjiang Medical University Guide for the Care and Use of Laboratory Animals.

Animals were randomly divided into 3 groups: control group (CTL, inhalation of cigarette smoke free room air [CS] exposure, n=10), CS exposure group (CS, n=10), CS exposure with lipopolysaccharide (LPS) infusion group (CS + LPS, n=10). Mice in the CS and CS+LPS groups were placed in a 60 x 40 x 30 cm^3^ fume box for 2h per exposure, twice daily, 6 days per week, and a mouse COPD model was established after 90 days of CS exposure (13). The AECOPD mouse model was established on day 91 by injection of LPS (1 μg/each, containing 50 μL of saline) (14). Equal amounts of saline were injected into the CS-exposed mice and the control group. Lung function, body weight and neutrophil counts in alveolar lavage fluid were assessed in all animals prior to execution on day 92. The cigarettes used in the mouse model establishment were commercial Honghe brand cigarettes (Hongyun Honghe Tobacco Industry, China). Each cigarette yields 10 mg tar, 1.0 mg nicotine and 11 mg CO. The total particulate matter concentration of 361.3 ± 49.2 μg/m^3^/day.

### 2.2 Hematoxylin and eosin (H&E) staining

Lung tissues were fixed in 4% formaldehyde for more than 24 h, paraffin-embedded, sliced and hematoxylin and eosin (H&E) stained. The sections were rinsed in running water, and then placed into acid water and ammonia water for color separation. After a few seconds, the sections were rinsed with running water for 1 h and then placed into distilled water. The slides were then immersed in 70% and 90% alcohol and dehydrated for 10 min. The slides were treated with alcohol eosin dye for 2 min, dehydrated, and sealed.

### 2.3 RNA extraction and quantitative polymerase chain reaction analysis

Total RNA was extracted from normal lung tissue, stable COPD lung tissue, and acute exacerbation COPD lung tissue (three biological replicates for each condition) using Trizol (Invitrogen, USA) following the manufacturer’s protocol. Total RNA was extracted using Trizol (Invitrogen, USA) following the manufacturer’s protocol. We used TaqMan^®^ RNA-to-CT™ 1-Step Kit from Thermo Fisher (METTL3, METTL14, FTO and YTHDF1) and PrimeScript RT Reagent Kit (MMP12, IL-1β, CXCL12, NFKBIA and CEBP-β) from Takara. Real-time quantitative PCR was performed on an ABI 7500 fast real-time PCR system (Thermo Fisher Scientific, USA) to determine the target RNA expression levels. The PCR system of TaqMan^®^ RNA-to-CT™ 1-Step Kit was as follows:48°C for 15 min, 95°C for 10min, followed by 40 cycles of 95°C for 15s, and 60°C for 1min. The PCR system of PrimeScript RT Reagent Kit was as follows: 95°C for 3 min, followed by 40 cycles of 60°C for the 30s, and 72°C for 10s. The relative quantity of the target gene was calculated with the 2^−△△Ct^ method and normalized GAPDH. The expression levels of METTL3, METTL14, FTO and YTHDF1 were assessed by TaqMan technology. Their primer sequence has not been listed because of intellectual property protection. However, other primer sequences are shown in **Table 1**.

**Table 1:** Primers of reverse transcription PCR analysis for genes.

Gene	Primer	Primer Sequence (5’-3’)
MMP12	Forward primer Reverse primer	AGAAGCAACTGGGCAACTGGAC CATCTTGACCTCTGGGGCACTG
IL-1β	Forward primer Reverse primer	CACTACAGGCTCCGAGATGAACAAC TGTCGTTGCTTGGTTCTCCTTGTAC
CXCL12	Forward primer Reverse primer	ACCAGTCAGCCTGAGCTACCG AAGGGCACAGTTTGGAGTGTTGAG
NFKBIA	Forward primer Reverse primer	CTGAAAGCTGGCTGTGATCCTGAG CTGCGTCAAGACTGCTACACTGG
CEBP-β	Forward primer	GCTGAGCGACGAGTACAAGATGC
	Reverse primer	CTTGTGCTGCGTCTCCAGGTTG
GAPDH	Forward primer Reverse primer	GAGAAGGCTGGGGCTCATTTGC TGCTGATGATCTTGAGGCTGTTGTC

### 2.4 MeRIP-seq

MeRIP-seq was performed by Novogene Technology Co., Ltd. (Beijing, China). Briefly, a total of 300 µg RNAs were extracted from the lung tissue. The integrity and concentration of extracted RNAs were detected using an Agilent 2100 bioanalyzer (Agilent, USA) and simpliNano spectrophotometer (GE Healthcare), respectively. Fragmented mRNA (~100 nt) was incubated for 2h at 4°C with anti-m6A polyclonal antibody (Synaptic Systems, USA) in the immunoprecipitation experiment. Then, immunoprecipitated mRNAs or Input was used for library construction with NEBNext^®^ Ultra™ RNA Library Preparation Kit for Illumina (New England Biolabs, USA). The library preparations were sequenced on an Illumina Novaseq or Hiseq platform with a paired-end read length of 150 bp according to the standard protocols. The sequencing was carried out with 3 independent biological replicates. The data has been uploaded to NCBI’s BioProject: https://www.ncbi.nlm.nih.gov/bioproject/PRJNA853736.

### 2.5 RNA-seq

RNA-seq was performed by Novogene Technology Co., Ltd. (Beijing, China). A total amount of 1 μg RNA per sample was used as input material for the RNA sample preparations. Briefly, mRNA was purified from total RNA using poly-T oligo-attached magnetic beads. Fragmentation was carried out using divalent cations under elevated temperature in First Strand Synthesis Reaction Buffer. First-strand cDNA was synthesized using a random hexamer primer and M-MuLV Reverse Transcriptase. Second-strand cDNA synthesis was subsequently performed using DNA Polymerase I and RNase H. Remaining overhangs were converted into blunt ends *via* exonuclease/polymerase activities. After adenylation of 3’ ends of DNA fragments, Adaptor with hairpin loop structure were ligated to prepare for hybridization. In order to select cDNA fragments of preferentially 370~420 bp in length, the library fragments were purified with the AMPure XP system (Beckman Coulter, Beverly, USA). Then PCR was performed with Phusion High-Fidelity DNA polymerase, Universal PCR primers, and Index Primer. At last, PCR products were purified and library quality was assessed on the Agilent Bioanalyzer 2100 system. The clustering of the index-coded samples was performed on a cBot Cluster Generation System using TruSeq PE Cluster Kit v3-cBot-HS according to the manufacturer’s instructions. After cluster generation, the library preparations were sequenced on an Illumina Novaseq platform and 150 bp paired-end reads were generated. The data has been uploaded to NCBI’s BioProject: https://www.ncbi.nlm.nih.gov/bioproject/?term=919075.

### 2.6 Data processing and statistical analysis

Adapters, duplicates and low quality sequences were removed from the raw reads and input samples of the IP using FASTP software to obtain clean data in fastq format. Me-RIP used BWA and RNA-seq used HISAT2 to compare the clean data to the small house mouse genome in Ensembl version 101 to obtain BAM files. Me-RIP uses exomePeak (Version: 2.16.0) software to analyze the distribution of peaks on the chromosomes and the differential genes in each group. Peak identification was performed for each experimental group with a threshold of FDR < 0.05 and Fold Enrichment > 1. Motif analysis we used the HOMER software to identify motifs on mRNA regions where m6A methylation occurred. RNA-seq using Stringtie software in results based on comparison to the genome on, the reads were spliced into transcripts and quantified, then analyzed for significant differences in expression using edgeR software. GO enrichment analysis was implemented by the GO seq R package. KEGG pathway analysis was implemented by KOBAS software (version 3.0). Correlation was analyzed using Pearson’s correlation. Peaks were visualized using IGV software and annotated with AI software. Weight, Neutrophil count and Gene expression levels of the samples were statistically analyzed using the Student t-test of GraphPad Prism 8 (GraphPad Software Inc, USA), and *P* < 0.05 was considered statistically significant.

## 3 Results

### 3.1 Construction of a mouse model of stable COPD and AECOPD and quantification of key m6A regulatory factors

**Figure 1:**
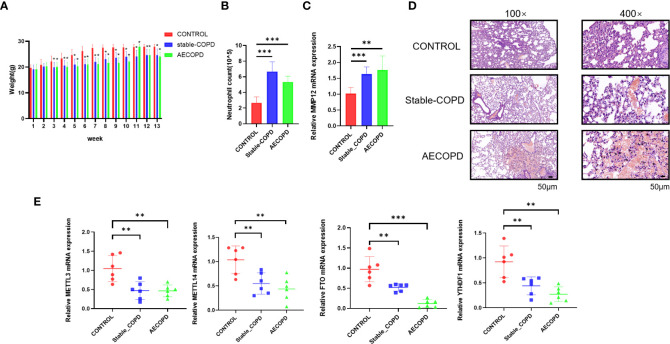
Detection of m6A methylation regulatory factor expression levels between the control and experimental groups: **(A)** Body weights of mice measured every week; **(B)** neutrophil count in the alveolar lavage fluid; **(C)** Detection of the expression level of MMP12 mRNA; **(D)** H&E staining of the lung tissue; **(E)** detection of expression levels of m6A methylation regulatory factor mRNAs (*P < 0.05; **P < 0.01, ***P < 0.001).

Analyses of weight, neutrophil count, and H&E staining can determine the successful establishment of this experimental model. The results showed that from the third week, the weights of the mice from stable COPD and AECOPD groups were significantly lower than those of the mice from the control group (**Figure 1A**). Further, the number of neutrophils in the alveolar lavage fluid was counted and analyzed by Giemsa staining. The neutrophil counts in stable COPD and AECOPD groups were higher than the count in the control group (**Figure 1B**). The RT-qPCR results showed that the expression levels of MMP12 mRNA were significantly higher in the stable COPD and AECOPD groups than in the control group (**Figure 1C**). H&E staining revealed the thinner and fused alveolar walls and immune cell infiltration into the alveoli of the mice from the stable COPD and AECOPD groups. These results demonstrate the successful construction of stable COPD and AECOPD mouse models (**Figure 1D**).

To explore whether m6A methylation participates in the occurrence and development of COPD, the expression levels of m6A methylation regulatory factors, including METTL3, METTL14, FTO, and YTHDF1, were compared by RT-qPCR. In comparison with the control group, stable COPD and AECOPD groups showed a significant decrease in METTL3, METTL14, FTO, and YTHDF1 levels (**Figure 1E**). Hence, one might assume that there are differences in m6A methylation during the occurrence and development of COPD, which will be further discovered through MeRIP-Seq.

### 3.2 Distribution difference of m6A methylation on chromosomes during the occurrence and development of COPD

**Figure 2:**
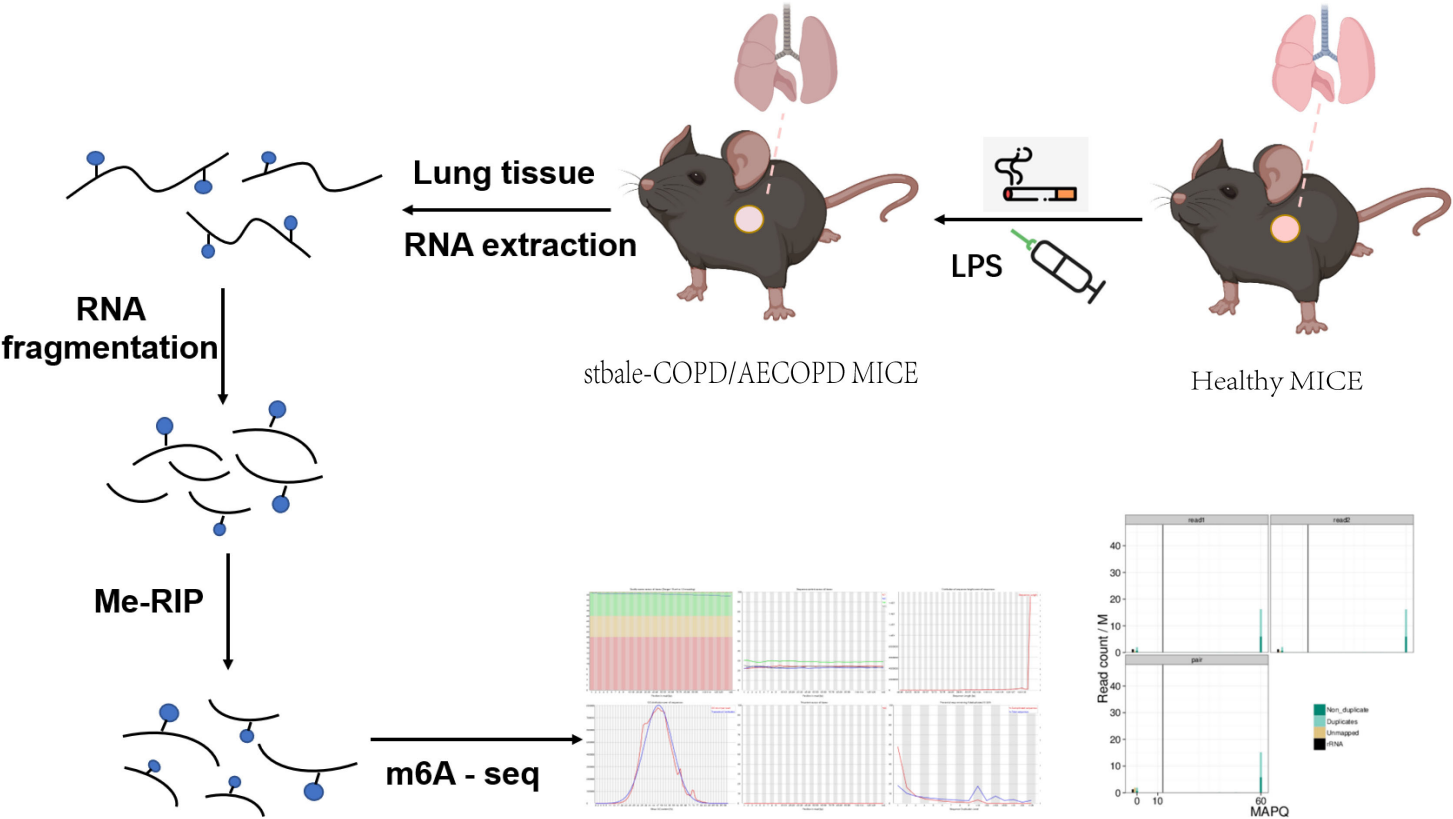
Schematic of m6A-seq analysis in C57 mice: C57 mice were stimulated with cigarette smoke or LPS, and their lung tissues were excised and used to extract total RNA. The RNA was fragmented and m6A RNA separated according to Me-RIP analysis. Construction of m6A-seq library and sequencing for analysis.

**Figure 3:**
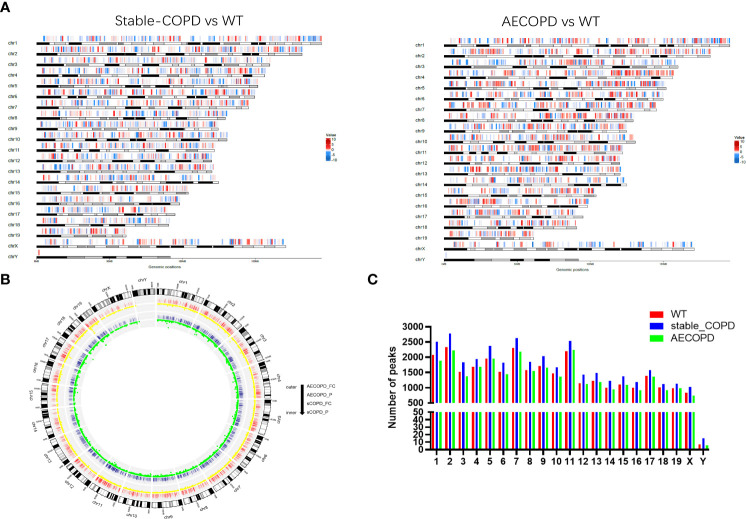
Distribution of differentially methylated m6A sites: **(A)** Distribution of all DMM sites in mRNAs and lncRNAs on chromosomes; **(B)** density distribution of m6A peak along chromosomes; **(C)** quantification of m6A peak on each chromosome. DMM: differentially methylated N6 methyladenosine.

We performed MeRIP-Seq to explore the role of m6A methylation in the occurrence and development of COPD (**Figure 2**). With three replicates and one INPUT per group, a total of 12 libraries were sequenced with m6A in the transcriptome range. All differentially methylated N6-methyladenosine (DMM) in mRNAs and long-noncoding RNAs (lncRNAs) were mapped to the chromosome to observe their distribution (**Figure 3A**). Analysis of the distribution of m6A methylation on different chromosomes showed that the chromosomes with the most m6A methylation were chromosome 2 (2338 m6A methylation peaks), chromosome 7 (2306 m6A methylation peaks), and chromosome 11 (2198 m6A methylation peaks) (**Figures 3B, C**). As shown in **Figure 3C**, in comparison with the control group, the stable COPD group had a significant increase and the AECOPD group had a significant decrease in the number of m6A methylation.

### 3.3 Overall m6A methylation modification on the transcriptome during the occurrence and development of COPD

**Figure 4:**
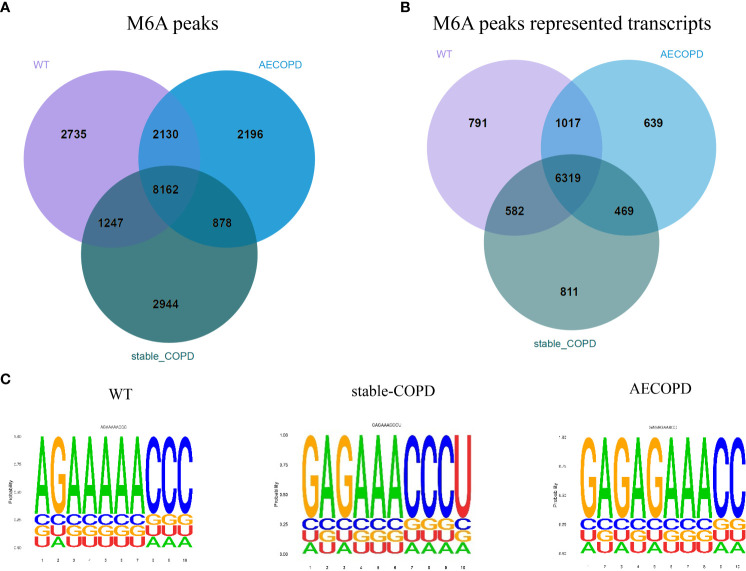
m6A-Seq of the transcriptome width reveals the overall m6A modification mode in the COPD group: **(A)** Number of common and specific m6A peaks in the control, stable COPD, and AECOPD group; **(B)** Venn diagram of transcript m6A peaks for the three groups; **(C)** motifs enriched from m6A peaks identified from the control, stable COPD, and AECOPD groups.

In the R package, A total of 14274 m6A peaks were detected for the control group, including 8709 gene transcripts, and 13231 m6A peaks were found for the stable COPD group, including 8181 gene transcripts. Similarly, 13366 m6A peaks corresponding to 8444 gene transcripts were observed for the AECOPD group. In addition, 8162 peaks that corresponded to m6A modification of 6319 genes were observed in three groups. The control, stable COPD, and AECOPD groups had their own 2735, 2944, and 2196 new peaks, respectively, which reflected significant differences in the overall m6A modification trend between them (**Figures 4A, B**). To determine whether the m6A peak contains an RRACH conservative sequence motif, samples from the three groups were tested and further analyzed by the HOMER software. The results showed that the consistent motif of m6A RRACH was AAACC (**Figure 4C**).

### 3.4 Distribution characteristics of transcriptome m6A methylation peaks during the occurrence and development of COPD

**Figure 5:**
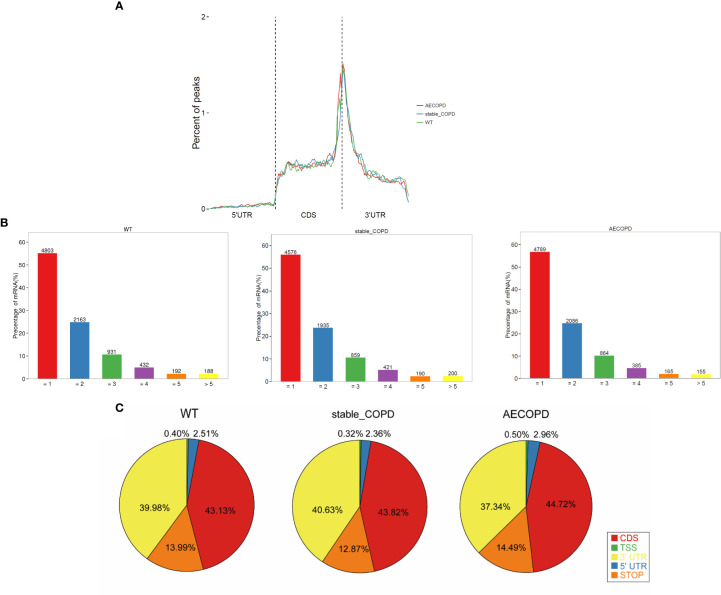
Distribution of m6A methyl groups in the transcript: **(A)** m6A peaks enriched with transcripts, wherein a transcript was separated into 5′-UTR, coding sequence, and 3′-UTR; **(B)** proportion of gene-processing m6A peaks with different numbers; **(C)** pie chart shows the percentage of m6A peak in the five segments of transcript. The m6A peak was most enriched in the CDS segment.

The meta-genomic model of the m6A peak was analyzed to determine the differential distribution of m6A in the full transcriptome. The results showed that the m6A peak was mainly concentrated at the beginning of the 3′-untranslated region (3′-UTR) (**Figure 5A**). Then, we explored the number of m6A modification peaks for each gene, and found that almost 60% of the methylated genes exhibited only one m6A peak while most genes contained 1-3 m6A peaks (**Figure 5B**).

To analyze the distribution of m6A peaks in mRNAs, we divided the transcript into five transcriptional fragments as follows: Transcription start site (TSS), 5′-untranslated region (5′-UTR), coding sequence (CDS), stop-codon, and 3′-UTR. The results demonstrated that m6A was most commonly detected in CDS and was also enriched in 3′-UTR (**Figure 5C**).

### 3.5 DEGs of m6A methylation during the occurrence and development of COPD

**Figure 6:**
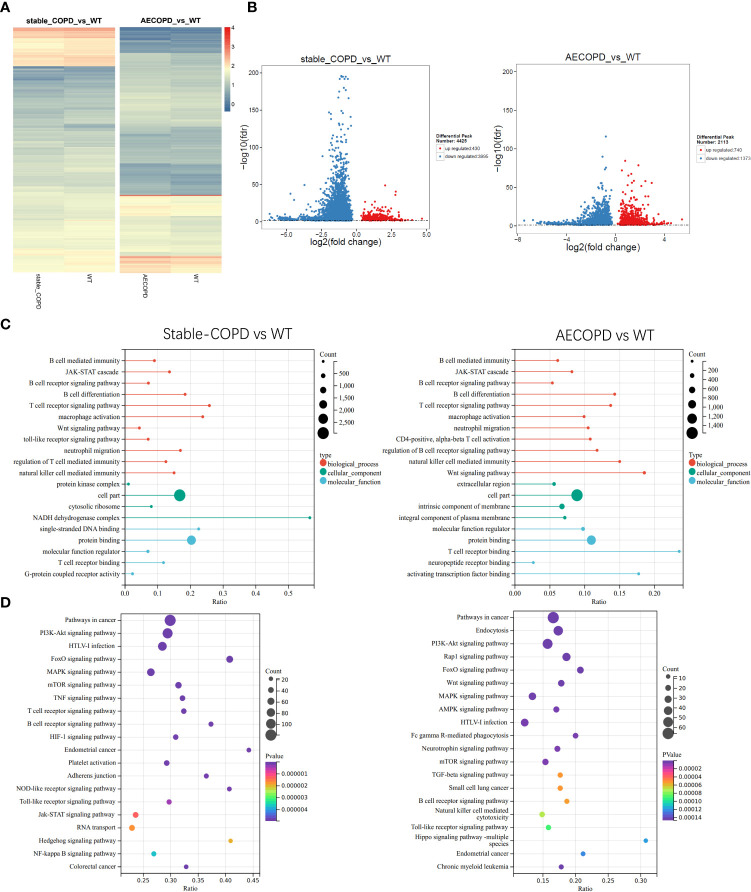
Gene Ontology and Kyoto Encyclopedia of Gene and Genomes analyses: **(A, B)** Heat map and volcano map show the m6A-modified differentially expressed genes (DEGs) between the control group and stable COPD and AECOPD groups. **(C)** GO terminologies of m6A-modified DEGs between the control group and stable COPD and AECOPD groups. **(D)** Twenty pathways of m6A-modified DEGs between the control group and stable COPD and AECOPD groups.

To explore the role of m6A in the occurrence and development of COPD, we compared the abundance of m6A peak between the stable COPD and control group as well as between AECOPD and control group. The stable COPD group had 430 upregulated and 3995 downregulated genes as compared with the control group. The top 10 upregulated and downregulated mRNAs are shown in **Table 1**. The AECOPD group has 740 upregulated and 1373 downregulated genes as compared with the control group. The top 10 upregulated and downregulated mRNAs are shown in **Table 2** (**Figures 6A, B**; **Tables 2**, **3**). Gene Ontology (GO) enrichment and Kyoto Encyclopedia of Genes and Genomes (KEGG) pathway analyses were used to investigate the differentially methylated genes (DMGs) and the biological significance of m6A methylation in the occurrence and development of COPD. GO analysis showed that during the occurrence and development of COPD, DMGs were mainly related to the regulation of Wnt signaling pathway, B cell receptor signaling pathway, natural killer cells regulating immunity, G-protein coupled receptor activity, and macrophage activation (**Figure 6C**). KEGG enrichment results showed the enrichment of different methylation genes in cancer pathway, PI3K signaling pathway, MAPK signaling pathway, and B cell receptor signaling pathway (**Figure 6D**). These results indicate that m6A methylation modifies many immune-related genes during the occurrence and development of COPD.

**Table 2:** Top 10 differentially expressed mRNAs between stable COPD and control group based on log_2_ (FC) value.

Genes	Gene Description	Chromosome	Peak Start	Peak End	P-value	Log_2(_FC)	Regulation
Col10a1	collagen, type X, alpha 1	10	34272424	34272604	2.69E-08	7.17	Up
Sirt1	sirtuin 1	10	63154903	63155053	1.17E-05	4.68	Up
Plin1	perilipin 1	7	79369965	79370206	9.12E-05	4.25	Up
0610030E20Rik	RIKEN cDNA 0610030E20 gene	6	72325914	72326064	1.32E-03	3.98	Up
Col10a1	collagen, type X, alpha 1	10	34271769	34271949	4.07E-03	3.98	Up
Cd37	CD37 antigen	7	44884338	44884757	1.02E-03	3.68	Up
Apoa1	apolipoprotein A-I	9	46141234	46141588	3.39E-03	3.53	Up
Dnah3	dynein, axonemal, heavy chain 3	7	119551373	1.2E+08	1.91E-03	3.49	Up
Mtres1	mitochondrial transcription rescue factor 1	10	43401247	43401367	4.57E-03	3.47	Up
Elovl6	ELOVL family member 6	3	129426730	1.29E+08	7.24E-03	3.46	Up
Ryr1	ryanodine receptor 1, skeletal muscle	7	28785386	28785717	1.12E-05	-8.34	Down
Clca1	chloride channel accessory 1	3	144710386	1.45E+08	1.32E-02	-7.8	Down
Lrrn4	leucine rich repeat neuronal	2	132721213	1.33E+08	4.17E-04	-7.42	Down
Mylk4	myosin light chain kinase family, member 4	13	32885258	32885618	6.61E-09	-7.13	Down
Ppfibp2	PTPRF interacting protein, binding protein 2	7	107347340	1.07E+08	1.86E-04	-6.64	Down
Gm15543	predicted gene 15543	6	145133106	1.45E+08	5.89E-05	-6.41	Down
Tango2	transport and golgi organization 2	16	18141792	18141973	5.25E-04	-6.21	Down
Kcnn3	potassium intermediate/small conductance calcium-activated channel	3	89578935	89579684	2.00E-12	-6.2	Down
Clca1	chloride channel accessory 1	3	144722441	1.45E+08	1.35E-04	-6.14	Down
Marveld1	MARVEL (membrane-associating) domain containing 1	19	42135838	42136078	1.07E-07	-6.1	Down

**Table 3:** Top 10 differentially expressed mRNAs between AECOPD and control group based on log_2_ (FC) value.

Genes	Gene Description	Chromosome	Peak Start	Peak End	P-value	Log_2(_FC)	Regulation
Tmem79	transmembrane protein 79	3	88236852	88237061	2.34E-05	3.68	Up
Zfp119a	zinc finger protein 119a	17	56173166	56173553	3.31E-04	3.66	Up
Zfp354c	zinc finger protein 354C	11	50705083	50706042	6.61E-04	3.56	Up
Dot1l	DOT1-like, histone H3 methyltransferase Symbol	10	80629380	80629949	1.00E-17	3.51	Up
Ncapg2	non-SMC condensin II complex, subunit G2 Symbol	12	116415950	116000000	1.32E-03	3.44	Up
BC024063	cDNA sequence BC024063	10	81946300	81947050	1.62E-03	3.42	Up
Gm12905	predicted gene 12905	4	123811429	124000000	3.80E-04	3.39	Up
Angel2	angel homolog 2	1	190660693	191000000	2.09E-05	3.38	Up
Zfp831	zinc finger protein 831	2	174487635	174000000	5.25E-03	3.25	Up
Zfp839	zinc finger protein 839	12	110834810	111000000	5.50E-03	3.24	Up
Obscn	obscurin, cytoskeletal calmodulin and titin-interacting RhoGEF Symbol	11	58903015	58903646	5.25E-09	-7.48	Down
Ighv4-1	immunoglobulin heavy variable 4-1	12	113911930	1.14E+08	1.00E-09	-6.76	Down
Cdr1	cerebellar degeneration related antigen 1	X	60228685	60228895	1.26E-05	-6.42	Down
Pde4dip	phosphodiesterase 4D interacting protein (myomegalin)	3	97597139	97597380	5.13E-05	-6.28	Down
Gm6712	predicted gene 6712	17	17538255	17539773	2.69E-07	-6.16	Down
Cdr1os	cerebellar degeneration related antigen 1, opposite strand	X	60228707	60228918	7.41E-05	-6.15	Down
Egln3	egl-9 family hypoxia-inducible factor 3	12	54226723	54228476	2.09E-07	-6.08	Down
Flnc	filamin C, gamma	6	29433284	29433495	1.26E-05	-5.84	Down
Thsd7a	thrombospondin, type I, domain containing 7A	6	12554958	12555199	6.61E-07	-5.82	Down
Kmt2a	lysine (K)-specific methyltransferase 2A	9	44714681	44714802	1.05E-04	-5.67	Down

### 3.6 Differentially expressed genes (DEGs) during the occurrence and development of COPD

**Figure 7:**
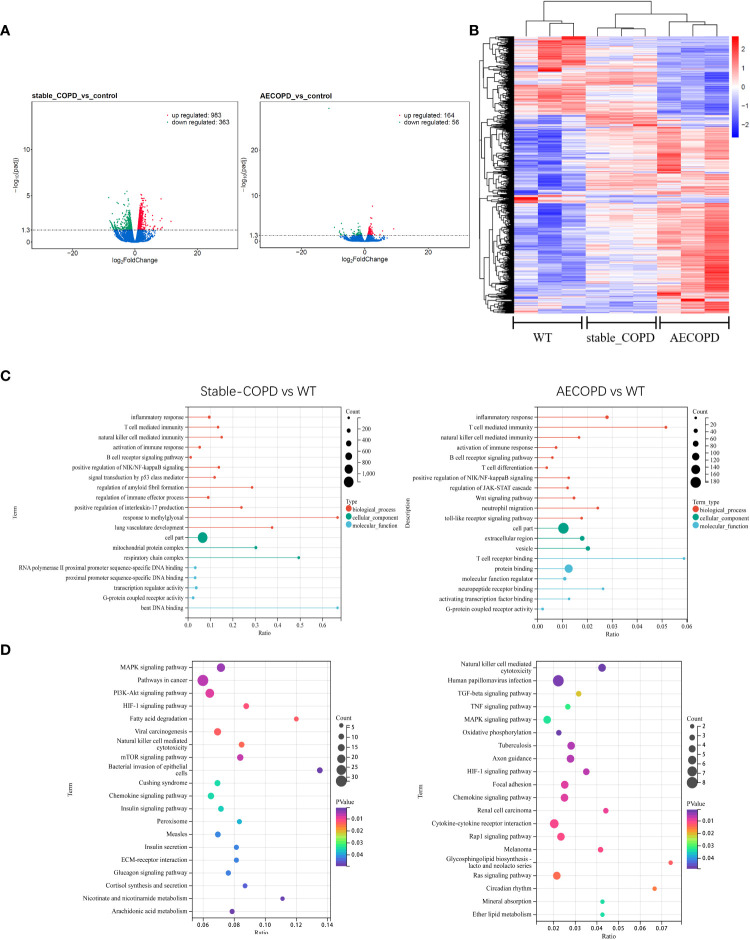
DEGs during the occurrence and development of COPD: **(A, B)** Volcano map and thermal map show DEGs in lung tissues (|log2FC |>1, *P* < 0.05). **(C)** GO enrichment analysis of DEGs. **(D)** KEGG enrichment analysis of DEGs.

To determine the expression profiles of DEGs in COPD lung tissues, DEGs between the control group and stable COPD and AECOPD groups were compared. The results showed 983 upregulated genes and 363 downregulated genes in the stable COPD group as compared with the control group. In comparison with the control group, the AECOPD group exhibited 164 upregulated and 56 downregulated genes (**Figure 7A**). For DEGs, the lung tissues from the same group showed a similar expression trend (**Figure 7B**). Further, GO and KEGG enrichment analyses were conducted for these DEGs. The results of GO enrichment analysis showed that these genes were related to functions such as immune response and regulation, including inflammation, activation of the immune response, B cell receptor signaling pathway, natural killer cell regulating immunity, T cell regulating immunity, NF-κB signal positive regulation, and G protein-coupled receptor activity (**Figure 7C**). KEGG analysis results revealed their involvement with a series of inflammation-related signaling pathways, such as natural killer cells-induced cytotoxicity, MAPK signaling pathway, HIF-1 signaling pathway (**Figure 7D**).

### 3.7 Joint analysis of m6A methylation and gene expression during the occurrence and development of COPD

**Figure 8:**
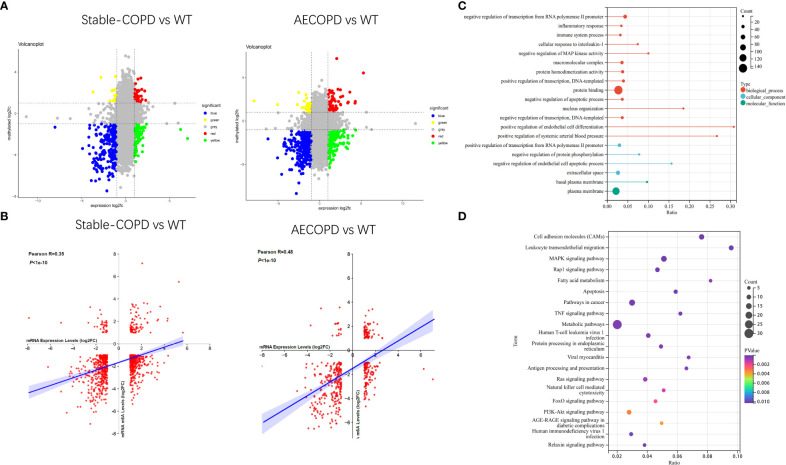
Joint analysis of MeRIP-seq and RNA-seq data: **(A)** Four quadrant graph shows the differentially methylated peaks in differentially expressed mRNAs (| log2FC>1, *P* < 0.05). **(B)** There was a positive correlation between differentially methylated mRNAs and the mRNA expression level (0.3<Pearson R <0.5, *P* < 1e-10). **(C, D)** GO and KEGG enrichment analyses of mRNAs with differential methylation and expression.

**Figure 9:**
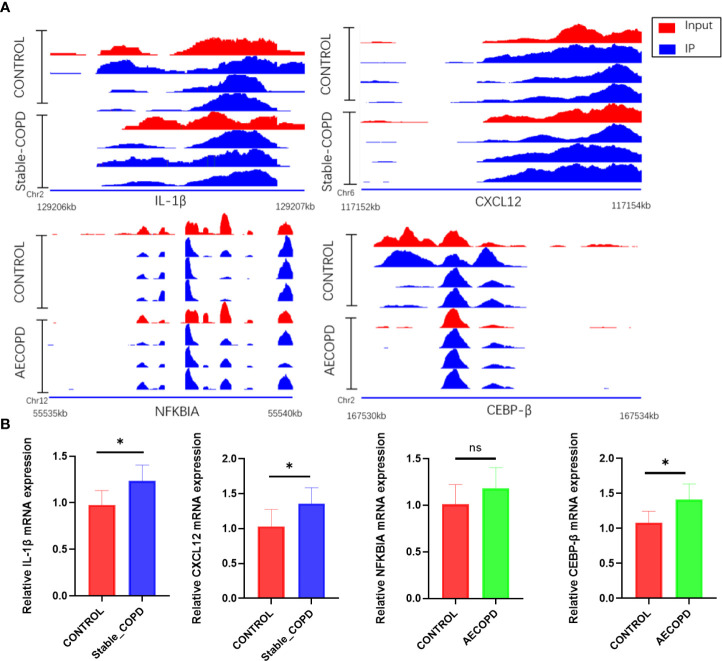
Changes in IL-1β, CXCL12, NFKBIA, and CEBP-β m6a modifications and mRNA expression levels during the development of COPD: **(A)** IGV tracks display the regional distribution of peaks in IL-1β, CXCL12, NFKBIA, and CEBP-β genes. **(B)** Detection of IL-1β, CXCL12, NFKBIA and CEBP-β mRNA expression levels by RT-qPCR (*P < 0.05). ns, P=0.2078.

MeRIP-seq and RNA-seq data were jointly analyzed and 986 and 445 differentially methylated mRNA transcripts were detected in the stable COPD and AECOPD group, respectively. Significant DEGs were found in these differentially methylated mRNA transcripts. In particular, 82 upregulated and 37 downregulated mRNAs were observed in the stable COPD group from 119 hypermethylated mRNAs and 419 upregulated and 448 downregulated mRNAs were obtained from 867 hypomethylated mRNAs. In the AECOPD group, 71 upregulated mRNAs and 16 downregulated mRNAs were obtained from 87 hypermethylated mRNAs and 115 upregulated and 243 downregulated mRNAs were detected from 358 hypomethylated mRNAs (**Figure 8A**). To clarify the relationship between methylation level and expression level of mRNAs, Pearson’s correlation analysis was carried out. The results showed a positive correlation between mRNA methylation level and mRNA expression level in both stable COPD and AECOPD groups (**Figure 8B**). This observation indicates that m6A methylation plays a key function in the regulation of gene expression in COPD. To determine the role of these differentially methylated mRNAs in the development of COPD, GO and KEGG enrichment analyses were conducted. These differentially methylated mRNAs were significantly enriched in GO terms such as protein-binding, inflammation, and immune system processes (**Figure 8C**). KEGG results showed that these differentially methylated mRNAs were significantly enriched in interleukin (IL)-17 signaling pathway, tumor necrosis factor (TNF) signaling pathway, natural killer cell-induced cytotoxicity, nuclear factor kappa B (NF-κB) signaling pathway, and other aspects, suggesting that m6A methylation may have an immunomodulatory effect in COPD (**Figure 8D**). Considering the pathogenesis of COPD involving immune disorders and excessive inflammation, it is necessary to explore whether there are mRNAs with m6A differential modification related to pathological process. We found many mRNAs related to immune regulation. Several representative mRNAs were detected, including IL-1β and CXCL12 in the stable COPD group and NFKBIA and CEBP-β in the AECOPD group. IGV was used to visualize the distribution of m6A modification in these mRNAs (**Figure 9A**). We examined the lung tissues of stable-COPD mice and AECOPD mice by RT-qPCR and showed that compared with the control group, the expression levels of IL-1β and CXCL12 mRNA were significantly increased in the stable-COPD group and CEBP-β mRNA in the AECOPD group, while the NFKBIA mRNA The differences in expression levels were not statistically significant (**Figure 9B**). The validation results were generally consistent with the predicted results.

## 4 Discussion

According to the World Health Organization, COPD is the third leading cause of death globally and costs are estimated to be in excess of 100 billion dollars per year (15, 16). COPD can be divided into stable COPD and AECOPD depending on the aggravation of clinical symptoms and its pathogenesis mainly involves inflammation (17). m6A, a dynamic RNA modification, is a key regulator of gene expression and plays an important role in the pathogenesis of various inflammatory diseases, and may also be involved in the development of COPD (18–22). Herein, we found that the expression levels of m6A key regulatory factors, namely, METTL3, METTL14, FTO, and YTHDF1 mRNAs, significantly decreased in the COPD group. In summary, m6A is involved in the occurrence and development of COPD. However, studies comprehensively examining m6A modification characteristics in COPD animal models and exploring its effects on the immune response in COPD are warranted. To supplement the existing literature, we performed MeRIP-seq and RNA-seq to reveal the m6A methylation characteristics of mice with COPD at the whole transcriptome level for the first time, and analyzed the methylation genes related to COPD and their potential functions in detail.

### 4.1 m6A methylation model of COPD

There were obvious m6A modifications in the lung tissues of normal and COPD mice. We found that the common RRACH sequence of m6A modification was AAACC in COPD through Homer; future studies should investigate the underlying reason. In addition, the enrichment of m6A modification in 3′-UTR was much higher than that in 5′-UTR, which was consistent with the previously reported distribution trend of m6A modification in lung tissues of acute allergic asthmatic mice. m6A usually appeared near the termination codon and 3′-UTR (23). High levels of m6A modification at 3′-UTR may be related to mRNA stability, selective polyadenylation, signal transduction, and translocation (24, 25). Further, m6A modification at 3′-UTR plays a regulatory role in protein translation by recruiting specific factors to these m6A modification sites for RNA transport or protein synthesis, which may be one of the reasons for the potential positive correlation between the degree of m6A methylation and level of transcription (26, 27). In this study, approximately 80% of methylation transcripts included one or two m6A peaks and 20% of m6A transcripts included three or more m6A peaks. Meanwhile, the distribution characteristics of the m6A peak were roughly the same in the three groups, and were mainly concentrated in the CDS and 3′-UTR areas. This observation is consistent with the peak distribution trend for peripheral nerve injury, allergic asthma, and rectal cancer reported in mice (28–30). In summary, these results show that m6A modification plays a role in COPD.

### 4.2 The relationship between differentially methylated mRNAs and airway inflammation in COPD

We observed several unique m6A peaks and methylated mRNAs in lung tissues of COPD mice at each stage. To determine the biological functions of differentially methylated mRNAs in COPD, GO and KEGG enrichment analyses were carried out. GO enrichment revealed the biological functions of most differentially methylated mRNAs in both stable COPD and AECOPD groups that were closely related to immune functions such as regulation of Wnt signaling pathway, B cell receptor signaling pathway, natural killer cell regulating immunity, G-protein coupling receptor activity, and macrophage activation. Previous studies have shown that the Wnt signaling pathway exerts immunomodulatory effects and is closely related to inflammation in COPD patients. For instance, the Wnt/β-catenin pathway promotes airway inflammation and Th17/Treg imbalance in COPD (31). Wnt4 can increase IL-8, IL-6, and monocyte chemoattractant protein-1 levels in bronchial epithelial cells, which may lead to neutrophil infiltration and inflammation in COPD (32, 33). Li et al. found that upregulation of Wnt signaling can weaken Toll-like receptor signaling and mediate inflammation of alveolar epithelial cells (34). KEGG enrichment results showed that the differentially methylated mRNAs were mainly enriched in the B cell receptor signaling pathway, Toll-like receptor signaling pathway, and other immune regulatory pathways. Janus kinase (JAK) signaling pathway and signal transducer and activator of transcription(STAT) signaling pathway participates in the occurrence of multiple inflammatory diseases and contributes to the pathogenesis of COPD by generating cytokines such as IL-1β and IL-6 (35). It is speculated that these differentially methylated mRNAs play a role in the pathogenesis of COPD by regulating inflammation-related pathways. m6A metabolism affects the stability and degradation of mRNAs and consequently regulates their expression (6, 36).

### 4.3 m6A methylation regulates gene expression

To investigate whether m6A modification affects gene expression in COPD patients, we evaluated differentially methylated loci gene expression. First, the gene expression spectrum was analyzed, and 983 upregulated and 363 downregulated genes were observed in the stable COPD group and 164 upregulated and 56 downregulated genes were detected in the AECOPD group. These DEGs participate in the immune regulation mechanism of COPD by modulating pathways such as inflammation, immune response activation, B cell receptor signaling pathway, and natural killer cells regulating immunity.

We jointly analyzed DEGs and DMGs in COPD lung tissues, and found that the stable COPD group had 82 upregulated hypermethylated mRNAs, 37 downregulated hypermethylated mRNAs, 419 upregulated hypomethylated mRNAs, and 448 downregulated hypomethylated mRNAs. The AECOPD group also showed 71 upregulated hypermethylated mRNAs, 16 downregulated hypermethylated mRNAs, 115 upregulated hypomethylated mRNAs, and 243 downregulated hypomethylated mRNAs. Pearson’s correlation analysis revealed a positive correlation between the methylation level of these genes and their expression levels in the two COPD groups. Downregulated hypomethylated mRNAs accounted for the largest proportion of COPD lung tissues. Hence, m6A methylation plays a dominant role in maintaining mRNA stability in COPD. Enrichment analysis revealed that some of these mRNAs are related to immune functions, including inflammation, immune system processes, IL-17 signaling pathway, and TNF signaling pathway. We selected several representative differentially methylated mRNAs from the above pathways (IL-1β, CXLC12, NFKBIA, CEBP-β), reviewed their roles in immunity and validated them by RT-qPCR.

As a member of the cytokine superfamily, the C-X-C motif chemokine ligand 12 (CXCL12) plays a chemotactic role in immunocytes. It was first found that CXCL12 exerts an important function in the regulation of proliferation and differentiation of pre-B cells. CXCL12 is closely related to inflammatory diseases and acts as a co-stimulator for the activation and proliferation of CD4^+^T cells in patients with chronic lymphoblastic leukemia. CXCL12 is also necessary for the differentiation of B-lymphoid and plasmacytoid dendritic cells (37, 38). IL-1β is an important inflammatory cytokine involved in the development of inflammation, and participates in the occurrence of various inflammatory diseases such as atherosclerosis, type II diabetes, and various autoimmune diseases (39). IL-1β also plays an important role in COPD by mediating the release of inflammatory substances such as colony-stimulating factors, cell adhesion factors, and hypersensitive response proteins (40). NFKBIA is a member of the NF-κB family that is closely related to head and neck, nasopharyngeal, and other cancers. It is a therapeutic target of small cell lung cancer (41–43). NFKBIA mutations promote the formation of IL-1β, and give rise to severe immune deficiency in liver diseases (44). A previous meta-analysis found that NFKBIA was closely related to autoimmune diseases and susceptibility to inflammatory diseases (45). As a transcription factor, CCAAT/enhancer-binding protein beta (CEBP-β) participates in the expression of inflammatory factors and the occurrence of various inflammatory diseases. It is one of the core genes of inflammation and infection prevention that can participate in the occurrence of COPD by regulating TNF-α and IL-17. Further, CEBP-β also participates in the mesenchymal transformation of bronchial epithelium by regulating IL-17 expression and thereby affecting the development of COPD (46, 47). Recent studies have shown that m6A reading protein IMP2 participates in the occurrence of autoimmune kidney injury by enhancing m6A modification of CEBP-β, promoting its stability, and upregulating the CEBP-β–mediated transcription of IL-17 (47). We then performed RT-qPCR on lung tissues from stable-COPD and AECOPD mice, and the results showed that compared with the control group, the expression levels of IL-1β and CXCL12 mRNA were significantly increased in the stable-COPD group and CEBP-β mRNA in the AECOPD group, while the NFKBIA mRNA The differences in expression levels were not statistically significant. The validation results were generally consistent with the predicted results. Refer to predictions, validation results and literature above, we speculate that m6A regulates the stability of RNAs such as CXCL12, IL-1β, and CEBP-β, increases their expression, and contribute to COPD.

## 5 Conclusions

A series of differentially methylated mRNAs that may be important regulators in COPD pathogenesis were determined. The first mouse m6A modification map in COPD lung tissues was drawn. This study provides a new direction for understanding the mechanism of m6A modification affecting inflammation and screening potential therapeutic targets for COPD.

## 6 Limitations

This study had potential limitations, although the target genes modified by m6A were known, the process of methylation readers, erasers or writers regulating the target genes was not delineated. Further studies should be conducted to investigate whether readers, erasers or writers regulate the stability, translation efficiency, or degradation of target genes. We found the above results in mice, but it is unclear whether the results are consistent in human lung tissue. In the future, we plan to collect samples from COPD patients and perform m6a-related assays on them.

## Data availability statement

The datasets presented in this study can be found in online repositories. The names of the repository/repositories and accession number(s) can be found below: PRJNA853736 and PRJNA919075 (NCBI Bioproject).

## Ethics statement

The animal study was reviewed and approved by Experimental Animal Ethics Committee of Xinjiang Medical University (approval no. SYXK-2018-0003).

## Author contributions

JW conceived and designed the experiments. TH, LX, MJ, FZ, QL, ZL, CW, JD, and FL performed the experiments. TH and LX analyzed the data. JW, TH, LX collected the fund. TH wrote the manuscript. MJ and JW revised the manuscript. All authors contributed to the article and approved the submitted version.
