# Supplementary material for: N6-methyladenosine-methylomic landscape of lung tissues of mice with chronic obstructive pulmonary disease
Source: Front Immunol. 2023 Mar 28;14:1137195. doi: 10.3389/fimmu.2023.1137195 (PMC10088907; doi:10.3389/fimmu.2023.1137195)
Supplement: Supplementary file 1 [file DataSheet_1.docx]

**1.1 DEGs of m6A methylation during the development of COPD**

We compared the abundance of m6A peaks between the stable-COPD group and the AECOPD group. The results revealed 3174 up-regulated and 907 down-regulated genes (FigS1.A-B). In addition, GO enrichment analysis and KEGG pathway analysis were used to analyze differentially methylated m6A genes, revealing the biological significance of m6A methylation during COPD development. GO analysis suggests that differentially methylated genes are primarily associated with immune system development, immune response, and T cell mediated immunity in the development of COPD (FigS1.C). Meanwhile, KEGG enrichment results showed that differentially methylated genes were enriched in PI3K-AKT signaling pathway, MAPK signaling pathway, and TNF signaling pathway (FigS1.D). These results suggest that m6A methylation modifies many immune-related genes during the development of COPD.


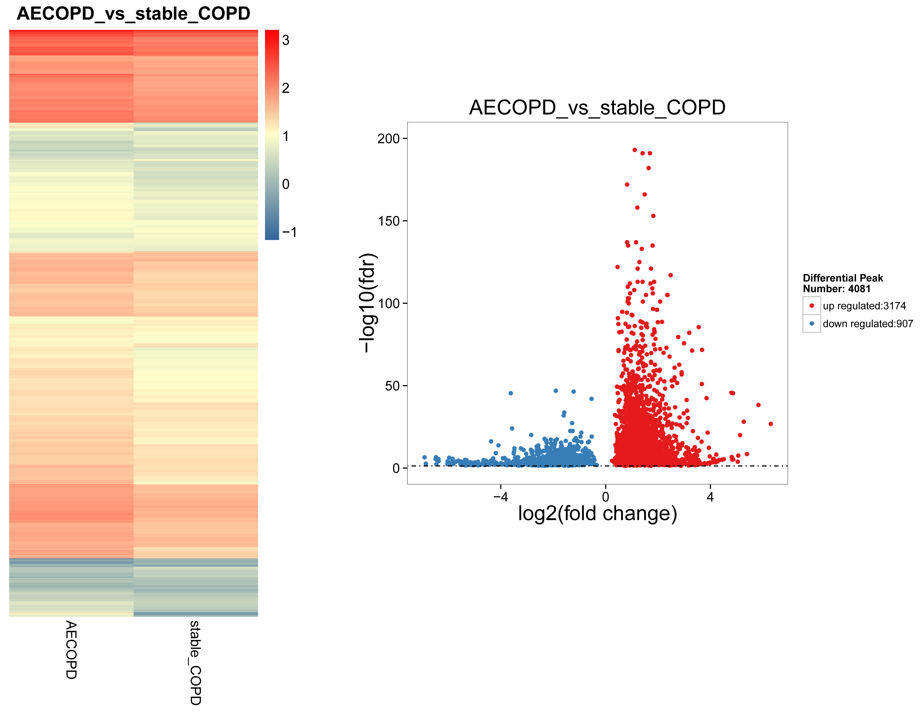


A

B


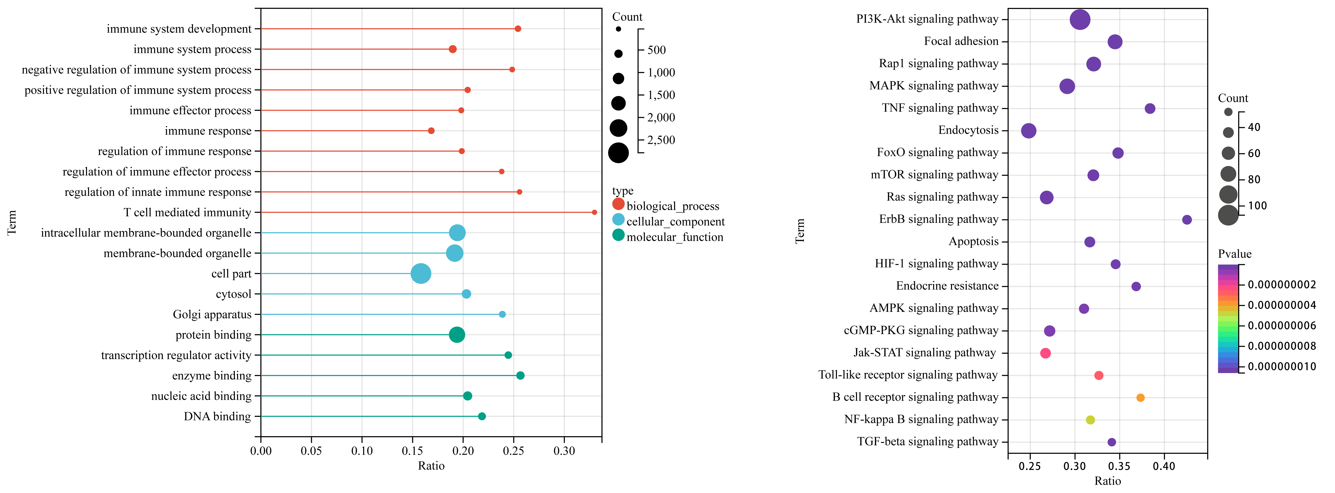


D

C

FigS1 Gene Ontology and Kyoto Encyclopedia of Gene and Genomes analyses: (A-B) Heat map and volcano map show the m6A-modified differentially expressed genes (DEGs) between stable COPD and AECOPD groups. (C) GO terminologies of m6A-modified DEGs between stable COPD and AECOPD groups. (D) Twenty pathways of m6A-modified DEGs between stable COPD and AECOPD groups.

**1.2 Differentially expressed genes (DEGs) during the development of COPD**

We also compared the differential gene expression between the stable-COPD and AECOPD groups. The results showed that 211 genes were upregulated and 251 genes were downregulated in the stable-COPD group compared to the AECOPD group (FigS2.A). We further performed GO and KEGG enrichment analysis on these differentially expressed genes. The results of GO enrichment analysis showed that these genes were related to functions such as immune response and regulation, including inflammation response, stimulatory killer cell immunoglobulin-like receptor signaling pathway, and Toll-like receptor 4 binding（FigS2.B）. KEGG analysis results revealed their involvement with a series of inflammation-related signaling pathways, such as PI3K-Akt signaling, IL-17 signaling pathways, and MAPK signaling pathways（FigS2.C）.


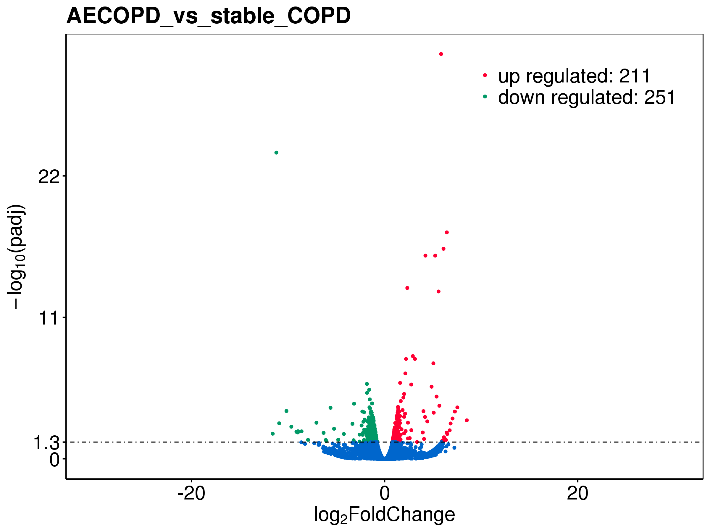


A


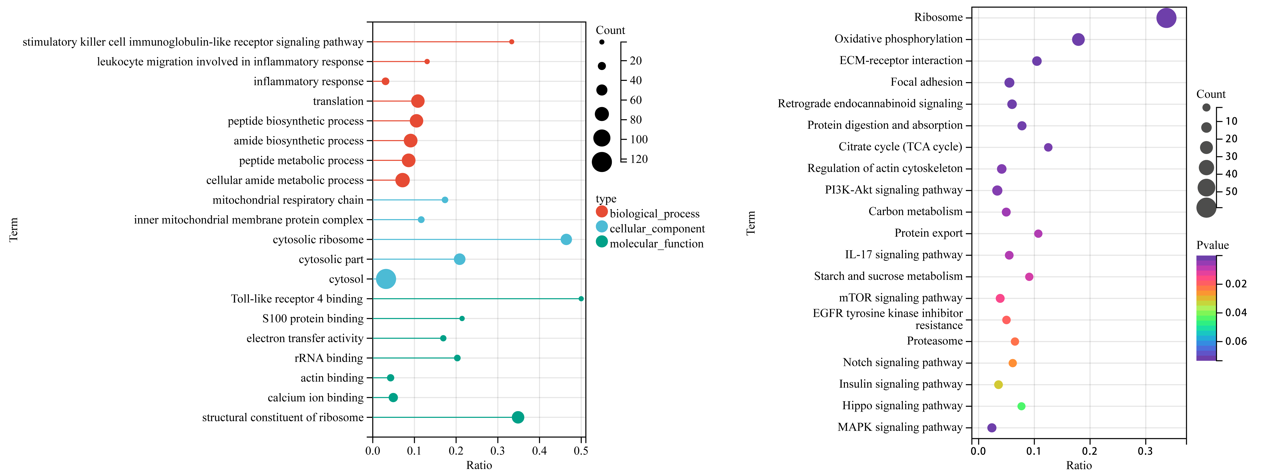


B

C

FigS2 DEGs during the development of COPD: (A) Volcano map show DEGs in lung tissues (|log2FC |>1, *P* < 0.05). (B) GO enrichment analysis of DEGs. (C) KEGG enrichment analysis of DEGs.
